# Alleviating Work Exhaustion, Improving Professional Fulfillment, and Influencing Positivity Among Healthcare Professionals During COVID-19: A Study on Sudarshan Kriya Yoga

**DOI:** 10.3389/fpsyg.2022.670227

**Published:** 2022-07-13

**Authors:** Divya Kanchibhotla, Prateek Harsora, Poorva Gupte, Saurabh Mehrotra, Pooja Sharma, Naresh Trehan

**Affiliations:** ^1^Sri Sri Institute for Advanced Research, Bangalore, India; ^2^Medanta – The Medicity, Gurgaon, India

**Keywords:** Sudarshan Kriya Yoga, healthcare professionals, professional fulfillment, work exhaustion, positive affect, negative affect, mental wellbeing

## Abstract

Demanding work-life and excessive workload, the conflict between professional and personal lives, problems with patients and those related to the occurrence of death and high risk for their own life are a few factors causing burnout, disengagement, and dissatisfaction in the professional lives of healthcare professionals (HCPs). The situation worsened during the COVID-19 pandemic. It is of utmost importance to find effective solutions to mitigate the stress and anxiety adversely affecting the mental well-being and professional lives of HCPs. This study was designed to examine the efficacy of Sudarshan Kriya Yoga (SKY) for alleviating work exhaustion, improving Professional Fulfillment, and influencing positivity among HCPs during COVID-19. In a comparative observation before the intervention (Pre), after the intervention (Post), and 30 days after the intervention (Day 30) in the Experimental Group (29 physicians) and Control Group (27 physicians), it was found that immediately after SKY, HCPs experienced a significant improvement in Professional Fulfillment (*p* = 0.009), work exhaustion (0.008), positive affect (*p* = 0.02), and negative affect (*p* < 0.001) compared to the Control Group. The effect of SKY continued until Day 30 for Professional Fulfillment and had positive and negative effects. Findings suggest that SKY elevated Professional Fulfillment among HCPs during the COVID-19 pandemic and reduced their work exhaustion and the negative effect on their mental health. SKY can aid HCPs in maintaining their well-being when faced with unprecedented challenges.

## Introduction

Since March 2020, there have been over 154.8 million confirmed cases of COVID-19 and more than 3,236,104 deaths worldwide (according to the World Health Organization, 2020). The already stretched medical and healthcare systems have significantly been strained due to the morbidity and mortality caused by COVID-19. Healthcare professionals (HCPs) have been at the forefront of dealing with the pandemic globally and are at a higher risk of exposure to COVID-19 (Nguyen et al., 2020). Stressful working conditions, longer working hours, and lack of available resources have contributed to anxiety, depression, work exhaustion, insomnia, and stress-related disorders among the HCPs during COVID-19 (Gupta and Sahoo, 2020; Kang et al., 2020; Pfefferbaum and North, 2020; Galbraith et al., 2021; Tiete et al., 2021). Supporting the health of HCPs and ascertaining wellness strategies that can be incorporated into their lifestyles is a critical need of the hour (Chirico et al., 2021).

A high prevalence of professional burnout, emotional exhaustion, and lower personal accomplishment was evident among HCPs even before the COVID-19 pandemic (Dyrbye et al., 2017; Elbarazi et al., 2017). Since the pandemic, their working challenges have been extreme. In a study, physicians were found to have compassion fatigue and burnout symptoms during COVID-19. The rate of burnout was higher in HCPs who worked in emergency and COVID-19 units (Ruiz-Fernández et al., 2020). The authors also found life satisfaction and resilience were inversely associated with COVID-19 related worries and rates of anxiety and depression (Barzilay et al., 2020; Duarte et al., 2020). Another study from India found that doctors were 1.64 times and support staff were five times more likely to experience pandemic burnout than the general population (Khasne et al., 2020). Similar results on the prevalence of burnout among HCPs were also reported from studies across the globe (Barello et al., 2020; Conti et al., 2021; Jalili et al., 2021; Martínez-López et al., 2021; O’Brien et al., 2021). A global survey found that the factors contributing to burnout among HCPs are mostly work impacting household activities, feeling pushed beyond training, being exposed to COVID-19 patients, and making life-prioritizing decisions (Morgantini et al., 2020). A study conducted during the COVID-19 pandemic among HCPs in India revealed that 3.7% of HCPs had high-level stress, 11.4% had severe depressive symptoms, and 17.7% had anxiety symptoms. This is comparable with the mental health of HCPs in other countries (Wilson et al., 2020). HCPs expressed a higher level of distress about the possibility of their family (48.5%) and other individuals with whom they come into contact (36%) getting exposed to COVID-19 than themselves (19.9%) (Barzilay et al., 2020). These challenges impact the quality of care, safety, and healthcare practice. Efforts are needed to address these growing challenges among HCPs (Dyrbye et al., 2017).

Delgado-Gallegos et al. (2020) conducted a study on HCPs and found a strong correlation between the number of COVID-19 patients seen per day and the traumatic stress experienced by the HCPs. COVID-19 has been found to be an independent risk factor for increasing mental health issues among HCPs during the pandemic (Spoorthy, 2020). This pandemic has had a highly adverse impact on the overall mental well-being of HCPs.

In a pre-pandemic study, Hilcove et al. (2020) and Ofei-Dodoo et al. (2020) found that mindfulness has a positive effect on the health and well-being of HCPs, as evidenced by pre and post measures of perceived stress, burnout, vitality, sleep quality, serenity, personal accomplishment, depression, anxiety, perceived resilience, and compassion. Based on such studies, Raudenská et al. (2020) also suggested implementing safety measures and appropriate interventions like mindfulness, cognitive behavioral therapy (CBT), imagination and trauma work, and gratitude practices to reduce occupational burnout and post-traumatic stress disorder (PTSD) among HCPs during COVID-19. However, these interventions have not been extensively studied during COVID-19 among HCPs, and further studies are required to support these findings (Amanullah and Ramesh Shankar, 2020).

Yoga and meditation improve the mental and physical well-being of an individual. Several studies on yoga and meditation have shown their benefits in reducing stress, anxiety, and depression (Balasubramaniam et al., 2013; Zollars et al., 2019; Park et al., 2020), improving sleep quality (Rusch et al., 2018; Guerra et al., 2020), and immunity (Nagarathna et al., 2020). Yoga has helped HCPs reduce burnout, emotional exhaustion, and depersonalization (Alexander et al., 2015; Abudurexiti et al., 2019).

Sudarshan Kriya Yoga (SKY) is a yogic breathing technique with a beneficial impact on stress, mental health, depression, sleep, social connectedness, and overall quality of life among youth, healthy adults, cancer, and HIV patients (Brown and Gerbarg, 2005; Kumar et al., 2013; Viveka et al., 2013; Mawar et al., 2015; Elbarazi et al., 2017; Seppälä et al., 2020). Further, SKY has been found to be helpful in improving the symptoms of anxiety and major depressive disorders and can be recommended as an adjunct treatment for depression and PTSD (Gangadhar et al., 1999; Brown et al., 2005; Doria et al., 2015; Sharma et al., 2017; Toschi-Dias et al., 2017). SKY decreases unpleasant sensations and blocked emotions (Kjellgren et al., 2007; Goldstein and Uchida, 2016), strengthening mental and physical well-being. A recent cross-sectional study on SKY during the COVID-19 pandemic demonstrated that SKY practitioners were able to maintain their mental health even during unusually stressful times (Parimala and Kanchibhotla, 2020). The current study investigates the role of SKY in alleviating Work Exhaustion and improving Professional Fulfillment among HCPs during COVID-19.

A vaccine for COVID 19 has been developed, and the first round of vaccination is underway. However, the question of how best to address the mental health issues arising from post-pandemic stress, economic recession, and fear of social interactions remains unresolved.

Under circumstances where the effectiveness of vaccination is not completely known, the timeline for regaining economic stability and creating COVID-19-free environments also remains unknown. It becomes imperative to find solutions to support HCPs on the frontlines to protect, maintain and boost their wellbeing. It is time to enlist and study every strategy available to us to help those responsible for society’s health. In such circumstances, it was hypothesized that SKY would be beneficial to mitigate the adverse effects of a pandemic like work exhaustion and disengagement among frontliners. This study was designed to observe the impact of SKY on alleviating work exhaustion, improving Professional Fulfillment, and influencing positivity among HCPs during COVID-19.

## Materials and Methods

### Study Design

A comparative observational study design was implemented among HCPs to observe the effect of SKY on the two groups: the Experimental Group and the Control Group. The assessments took place between July 2020 and October 2020. The study was conducted online, keeping in mind HCPs’ busy schedules and isolation protocols due to COVID-19 patient care. Conducting an interview or applying mixed methods in study design was not possible due to their work prioritization. The subjects in the Experimental Group were assessed at three time points: before the intervention (Pre), after the intervention (Post) on Day 4 to observe the immediate effects of the intervention, and 30 days after the daily practice of the intervention (Day 30). The Control Group was assessed on the first day of the study and had received no intervention. A team that was not directly involved in the study collected data at Medanta to reduce demand characteristics. The data were collected utilizing standardized questionnaires on resilience, Professional Fulfillment, and mood from HCPs who underwent an online SKY Workshop. The study was carried out in collaboration with two institutions—Sri Sri Institute for Advanced Research and Medanta Institute of Education and Research (MIER). The Medanta Institutional Ethics Committee approved this study (reference number MICR-1106/2020).

### Participants

Study participants were recruited from Medanta Hospital. The institute is located in Sector 38, Gurgaon, Haryana, India. During the pandemic, Medanta was one of the foremost institutions taking care of COVID-19 patients in Delhi NCR, the capital city of India. The inclusion criteria for study participants were direct exposure to COVID-19 patients, no previous experience of SKY, and a willingness to participate in the study. The doctors practicing in the study field from July 2020 to October 2020 were included in the study. All the working professionals from Medanta were called and informed about the details of the study over the phone. They were also informed that their participation in the study was voluntary. Those who gave voluntary consent to participate in the study were included, and their informed consent was obtained. The institutional review board of Medanta approved the study. Pregnant women and those unable or unwilling to practice SKY were excluded from the study. There was no compensation or cost to participate in this study, and the medical records and personal information of the HCPs were kept confidential.

**FIGURE 1:**
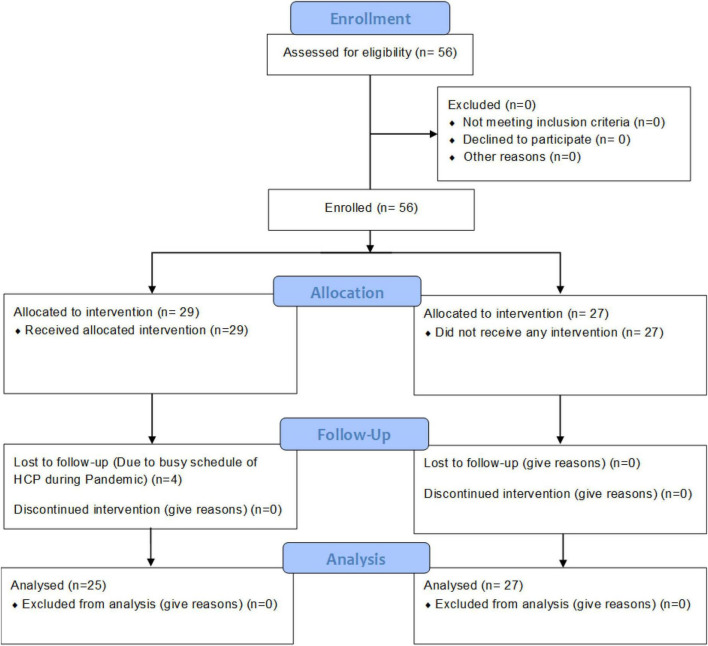
Flow diagram.

The study was conducted during the peak of the first wave in India. The subjects were healthcare providers who were actively tending to COVID-19 patients; hence, their time and availability were under severe constraints. All study participants attended SKY workshops from their place of comfort, either an office, clinic, COVID center, or their home. However, most of the study participants were staying near COVID care centers. SKY workshops were conducted online *via* Zoom. There was no in-person social contact during the workshop or during the study duration due to the SKY intervention. The workshops were limited to 15 participants and were led by experienced yoga instructors. Each session was led by two trainers to ensure individual attention. The workshop lasted for 4 days, 2 h daily. The Experimental Group attended only SKY sessions due to their hectic schedule and did not participate in any other forms of yoga, meditation, or physical or social activities. In total, 4–5 workshops were conducted over 2 weeks for all study participants. Study participants were allowed to join either the morning, afternoon, or evening batch, depending on their preference and availability. After completion, regular follow-up sessions were conducted every morning and evening for 30 days, maintaining attendance logs. A follow-up call was made once a week to enhance retention. In the Experimental Group, a total of 29 HCPs contributed to pre- and post-study assessments. Three participants were excluded from the study due to their inability to practice SKY for the entire 30 days, leaving 26 of them to participate on Day 30. From the Control Group, 27 HCPs participated; however, it was impossible to provide them with any wellness program or treatment due to their busy schedule and the pandemic. The flow diagram of study is represented in the Figure 1.

### Intervention

Sudarshan Kriya Yoga is a method of cyclical controlled breathing and meditation practice. A typical SKY session lasts for 30 m and consists of four distinct yogic breathing stages (Ujjayi, Bhastrika, Om, and Sudarshan Kriya). Ujjayi involves becoming aware of the conscious sensation of the breath touching the throat. This slow breathing technique is done at a rate of 2–4 breaths per minute (bpm). During Bhastrika, the air is rapidly inhaled and forcefully exhaled at a rate of 20 bpm. Three 1-min rounds of Bhastrika are followed by a few minutes of normal breathing. Next, Om is chanted three times with very prolonged expiration. Lastly, Sudarshan Kriya rhythmic breathing is performed at three different speeds: slow (20 bpm), medium (40–50 bpm), and fast (60–80 bpm). A SKY session is conducted in a seated position with the eyes closed.

Sudarshan Kriya Yoga was taught to participants in a 4-day online breathing and meditation workshop. The duration of the workshop was a total of 8 h (a 2-h session each day) and was taught by video conference. The workshops were led by experienced SKY instructors trained by the Art of Living Foundation. The participants were given home practice and were encouraged to practice daily. The home practice was 25 m long.

### Study Measures

The Stanford Professional Fulfillment Index is a 16-item Professional Fulfillment scale that assesses the degree of intrinsic positive reward an individual receives from their work, including happiness, meaningfulness, contribution, self-worth, satisfaction, and feeling in control when dealing with difficult problems at work. It is divided into three domains: Professional Fulfillment, work exhaustion, and interpersonal disengagement. Responders were asked to rate every item on a 5-point Likert scale (“not at all true” to “completely true” for Professional Fulfillment items and “not at all” to “extremely” for work exhaustion and interpersonal disengagement items). For the domain of Professional Fulfillment, higher scores indicated higher Professional Fulfillment. For the domain of Work Exhaustion, higher scores indicated greater Exhaustion, and for Interpersonal Disengagement, a higher score indicated greater interpersonal disengagement. Test-retest reliability estimates 0.80 for overall burnout (α = 0.92). This 16-item small scale measured both burnout and Professional Fulfillment and was developed for use among physicians in any healthcare setting. It is sensitive, reliable, and not time-consuming to complete. Compared with the Maslach Burnout Inventory, the PFI burnout scale sensitivity and specificity in identifying those with burnout were found to be 72 and 84%, respectively (Shanafelt et al., 2019).

Positive and Negative Affect Scale (PANAS) (Watson et al., 1988): positive affect (PA) reflects the extent to which a person feels enthusiastic, active, and alert. In contrast, negative affect (NA) is a general dimension of subjective distress and unpleasurable engagement. PANAS is a 20-item scale rated on a 5-point Likert scale (1 = not at all to 5 = extremely). High PA is a state of high energy, concentration, and pleasurable engagement, whereas low PA is characterized by sadness and lethargy; it is associated with calmness. This is the oldest, most reliable, and validated scale, and it is easy to understand and use. The reliability of the PANAS was found to be high, and the pattern of relationships between the PANAS and the measures of depression and anxiety (the HADS and the DASS) was found to be consistent with tripartite theory (Crawford et al., 2004).

Both the standardized tools mentioned above have been used in several studies in India and are highly used in yogic interventional studies.

The study population was frontline workers with busy schedules. Keeping in mind the critical nature of the HCPs’ work and the associated paucity of time, we kept the time for assessments to a minimum. Accordingly, PFI and PANAS were found suitable for this study. Due to the aforementioned reasons, other research methodologies such as interviews and group discussions were not found to be suitable for the study population.

### Data Analysis

Participants from the Experimental (SKY) group were assessed at three time points: pre, post, and day 30. A team not directly involved in the study collected data at Medanta to reduce demand characteristics. Data from the Experimental and Control Groups were compared using two-way MANOVA at a significance level of 0.05. For the Experimental Group, data were matched in pairs of pre–post, pre-day 30, and pre–post-day 30. Sample size analysis was performed using G-Power. Data were recorded in Microsoft Excel, and analyses with the appropriate graphical presentation were performed using the IBM Corp. Demographic variables are tabulated and graphically represented using proportions (%). The Chi-square or Fisher’s exact tests were used to compare groups for categorical data. Wherever applicable, quantitative data are presented with mean and standard deviation (SD) and analyzed using the Student’s *t*-test (paired and unpaired). Pearson Correlation was calculated in the Experimental Group to find the linear correlation between the two variables. Cronbach’s alpha was used to determine the internal consistency and reliability of the data, while Cohen’s *d* value was used to determine the effect size within the Experimental Group.

## Results

**FIGURE 2:**
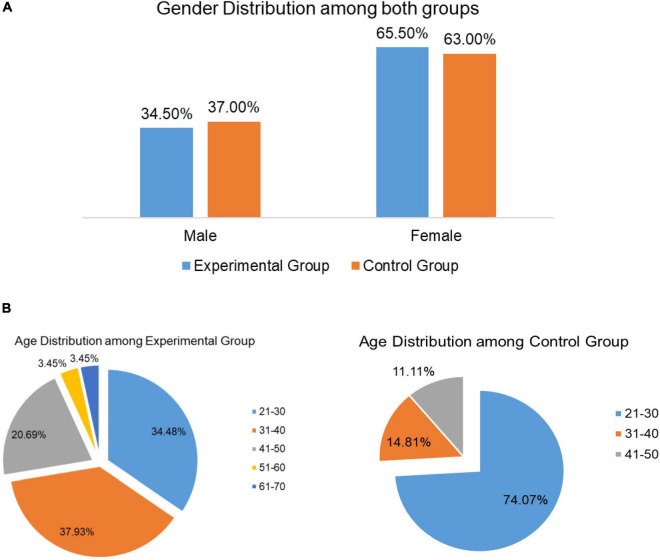
**(A)** Gender distribution in the Experimental and Control Groups. **(B)** Age distribution in the Experimental and Control Groups.

Demographic details for the SKY and Control Group are shown in Table 1. Both groups had comparable populations. 65.5 and 63.0% of the population were females in the SKY and Control Groups, respectively, as shown in Figure 2A. The Experimental Group had participants in the age group of 25–66, while those in the Control Group ranged in age from 21 to 47 (Figure 2B).

**TABLE 1:** Participants’ characteristics at baseline for the Experimental Group (*N* = 29) and Control Group (*N* = 27).

Characteristics	Type	All participants in the Experimental Group	All participants Control Group
Gender *n* (%)	Male	10 (34.50)	10 (37.00)
	Female	19 (65.50)	17 (63.00)
Age	Mean (SD)	37.1 (9.90)	28.5 (7.30)
	Min	25	21
	Max	66	47

Tables 2–4 depict the average values between the Experimental and Control Groups at three different time points: Pre, Post, and Day 30, respectively, for each domain. Before the intervention, the two groups did not differ significantly on any outcome (Table 2).

**TABLE 2:** Average values (standard deviation) comparison between Experimental and Control Groups before the intervention.

	PRE Experimental	PRE Control	Pre Experimental_Pre Control
	Mean (SD)	Mean (SD)	*P*-value
Professional Fulfillment	17.48 (5.24)	14.96 (6.10)	0.28
Work exhaustion	5.16 (3.70)	5.81 (3.64)	0.44
Interpersonal disengagement	5.00 (5.67)	4.40 (5.38)	0.94
PANAS positive	36.92 (9.08)	36.33 (9.22)	0.84
PANAS negative	19.80 (7.12)	24.22 (7.88)	0.06*

*p-Values < 0.05 and **p-value < 0.01.

*p-Values are based on two-way MANOVA at the significance level of 0.05.*

**TABLE 3:** Average values (standard deviation) comparison between Experimental and Control Groups after the intervention.

	POST Experimental	POST Control	Post Experimental_Post Control
	Mean (SD)	Mean (SD)	*P*-value
Professional Fulfillment	19.12 (4.87)	14.96 (6.10)	0.04*
Work exhaustion	3.28 (2.91)	5.81 (3.64)	0.01*
Interpersonal disengagement	3.96 (6.40)	4.4 (5.38)	0.62
PANAS positive	41.88 (7.20)	36.33 (9.22)	0.05*
PANAS negative	15.52 (7.89)	24.22 (7.88)	0.00**

*p-Values < 0.05 and **p-value < 0.01.

p-Values are based on two-way MANOVA at the significance level of 0.05, *p-values < 0.005 and **p-value < 0.001.

**TABLE 4:** Average values (standard deviation) comparison between Experimental and Control Groups 30 days after the intervention.

	Day 30 Experimental	Day 30 Control	Day 30 Experimental_Day 30 Control
	Mean (SD)	Mean (SD)	*P*-value
Professional Fulfillment	19.00 (5.12)	14.96 (6.10)	0.02*
Work exhaustion	4.52 (3.86)	5.81 (3.64)	0.20
Interpersonal disengagement	2.36 (3.35)	4.40 (5.38)	0.05*
PANAS positive	41.60 (7.14)	36.33 (9.22)	0.05*
PANAS negative	14.96 (6.06)	24.22 (7.88)	0.00**

*p-Values < 0.05 and **p-value < 0.01.

p-Values are based on two-way MANOVA at the significance level of 0.05, *p-values < 0.005 and **p-value < 0.001.

**FIGURE 3:**
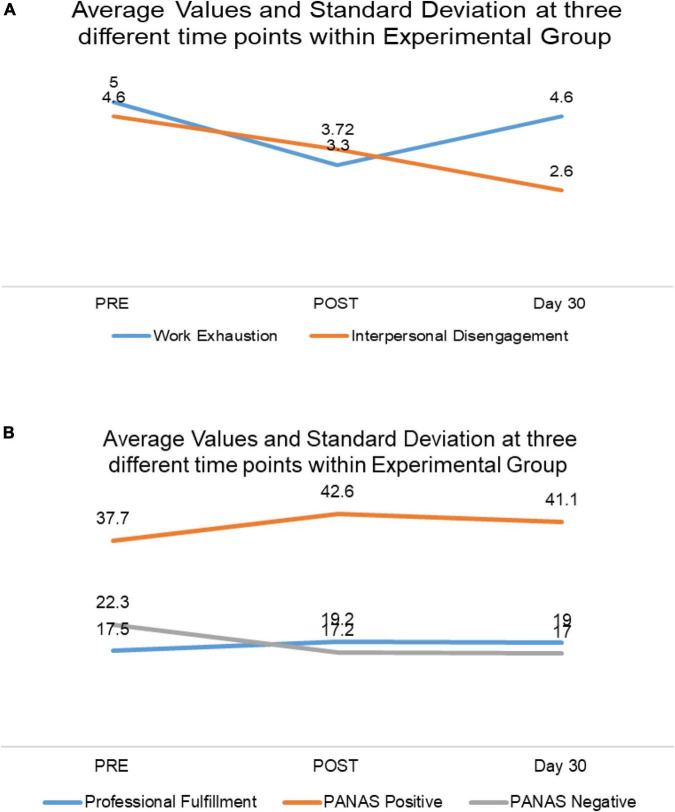
**(A)** Average values and standard deviation at different study time points within the Experimental Group. **(B)** Average values and SD at different study time points in the Experimental Group.

Using G-power, the effect size was 0.76 (medium effect size) when alpha was 0.05, and the power of the study was 80% (Faul et al., 2007). Table 5 depicts the effect size using Cohen’s *d* value for different domains in the SKY group, indicating small-to-large size effects for different domains. Figures 3A,B show the variation of dependent variables in the Experimental (SKY) Group at three different time points.

**TABLE 5:** Effect size in the Experimental Group.

	Cohen’s *d* value	
	pre_post	pre_day 30
Professional Fulfillment	−0.36	−0.32
Work exhaustion	0.51	0.14
Interpersonal disengagement	0.15	0.53
PANAS positive	−0.62	−0.50
PANAS negative	0.60	0.70

There was a statistically significant difference between the two groups when compared at three different time points, once pre and twice post-intervention [*F*(16,33) = 5.3, *p* < 0.001; Wilk’s λ = 0.28, partial η^2^ = 0.72]. There was no statistically significant difference observed between the males and females or interaction between the group and gender when compared at three different time points.

### Professional Fulfillment

At the baseline assessment, SKY and Control scored similarly for Professional Fulfillment. After practicing SKY, the HCPs in the Experimental Group scored significantly higher in the Professional Fulfillment domain than those in the Control Group [*F*(1,48) = 4.51; *p* < 0.05; partial η^2^ = 0.09]. The mean value increased from 17.5 to 19.2 in the Experimental Group immediately after the intervention. A similar effect was observed on the 30-day assessment [*F*(1,48) = 5.43; *p* < 0.05; partial η^2^ = 0.10].

Excellent internal consistency was demonstrated with Cronbach’s alpha α ≧ 0.9 at all the three-time assessment points. The effect size of the intervention for the Experimental Group was −0.4 for pre–post and −0.3 for pre-day 30. This indicated a medium-sized effect.

### Work Exhaustion

After experiencing SKY, work exhaustion was significantly reduced among the HCPs from the Experimental Group compared to those from the Control Group [*F*(1,48) = 7.74; *p* < 0.05; partial η^2^ = 0.14]. Average values dropped from 5.00 to 3.30. This effect did not last until Day 30, probably because of increased workload and a steady increase in the number of COVID-19 patients.

Cronbach’s alpha demonstrated good internal consistency with 0.8 ≧ α ≧ 0.7 at all the three-time assessment points. The effect size of the intervention for the Experimental Group indicated a small-to-medium-sized effect (Table 5). According to Bennett et al. (2021), 69% of the population experienced improved work exhaustion immediately with the SKY intervention and 58% at 30 days after the intervention.

### Interpersonal Disengagement

The interpersonal disengagement score did not differ significantly when compared with that of the Control Group post-intervention; however, it differed significantly on Day 30 [*F*(1,48) = 3.95; *p* < 0.05; partial η^2^ = 0.08] when an intragroup comparison was performed in the SKY group; it was found that interpersonal disengagement scores reduced significantly with the following values: pre-mean: 4.60; post-mean: 3.72; day 30 mean: 2.60, *p*-value: 0.51 (Pre and Post), and *p*-value: 0.02 (Pre-day 30) (data shown in Table 6).

**TABLE 6:** Average values (standard deviation) in the Experimental Group.

	PRE Experimental	POST Experimental	Day 30 Experimental	*P*-values Experimental	
	Mean (SD)	Mean (SD)	Mean	pre_post	pre_day 30
Professional Fulfillment	17.50 (5.1)	19.20 (4.7)	19.00 (5.0)	0.06	0.10
Work exhaustion	5.00 (3.6)	3.30 (3.0)	4.60 (3.8)	0.01**	0.20
Interpersonal disengagement	4.60 (5.4)	3.72 (6.1)	2.60 (3.5)	0.51	0.02*
PANAS positive	37.70 (8.8)	42.60 (7.0)	41.10 (7.3)	0.01*	0.07
PANAS negative	22.3 (8.2)	17.20 (8.8)	17.0 (6.7)	0.00**	0.00**

*p-Values < 0.05 and **p-value < 0.01.

p-Values are based on paired sample t-test at the significance level of 0.05, *p-values < 0.005 and **p-value < 0.001.

The intervention effect size for the Experimental Group was 0.2 for pre–post and 0.5 for pre-day 30. This indicated a small-sized effect immediately after intervention and a medium-sized effect after 30 days of SKY practice. According to Bennett et al. (2021), it signifies that 58 and 69% of the population experienced significant improvement in their interpersonal disengagement immediately with the SKY intervention and 30 days after the intervention, respectively. Cronbach’s alpha demonstrated excellent internal consistency with α ≧ 0.9 at all three-time assessment points.

### Positive and Negative Affect

The SKY group showed significant improvement in positive and NA post-intervention compared to the Control Group: [*F*(1,48) = 4.03; *p* < 0.05; partial η^2^ = 0.08] for PA and [*F*(1,48) = 14.10; *p* < 0.05; partial η^2^ = 0.23] for NA. The same effect was observed at the Day 30 assessment: [*F*(1,48) = 3.82; *p* < 0.05; partial η^2^ = 0.07] for PA and [*F*(1,48) = 19.25; *p* < 0.05; partial η^2^ = 0.29] for NA. The PA mean value increased from 37.7 to 42.6. Immediately after the intervention, the NA mean value in the Experimental Group decreased immediately from 22.3 to 17.2 and 17.0 on day 30.

For positive and NA, Cronbach’s alpha demonstrated excellent internal consistency with α > 0.9 at all three-time assessment points. For the SKY group, the effect size of the intervention varied from −0.5 to −0.7 for pre–post and pre-day 30, indicating a medium-sized effect for both domains (Table 5). According to Bennett et al. (2021), it signified that 73% of the population experienced significant improvement in their positive and NA immediately after SKY intervention. While 69% and 76% of the population experienced significant improvement in PA and NA respectively, after 30 days of SKY intervention.

## Discussion

The COVID-19 pandemic has been a crucial time for science. Impressive efforts have been made to develop a vaccine against COVID-19 and to investigate the pandemic’s impact on the physical and mental health of human beings. HCPs were found to be the most affected by the pandemic due to increased stress and work exhaustion, which, in turn, affected their quality of life (Çelmeçe and Menekay, 2020; Pearman et al., 2020). Many have also suggested implementing mental health wellness programs and strategies among HCPs (Cullen et al., 2020; Petzold et al., 2020; Pfefferbaum and North, 2020; Chirico et al., 2021; Martínez-López et al., 2021; O’Brien et al., 2021; Tiete et al., 2021). This research study moves one step further to investigate whether yoga can provide a solution to address the adverse effect of COVID-19 on the mental health of frontline workers. HCPs are the backbone of a healthy society. Therefore, this study aims to shed light on methods to assist HCPs in maintaining their physical and mental well-being and, through helping the HCPs, also to strengthen the health of every individual in our society—not just during the COVID-19 pandemic but also during the new “normal” that will emerge post-pandemic. Numerous studies have shown the efficacy of yoga-based mind-body interventions such as SKY in improving mental and physical health, not only in everyday life but also in extremely stressful situations such as prison incarceration (Seppälä et al., 2014; Deuchar, 2019), natural or man-made disasters (Descilo et al., 2010), and conflicts (Kanchibhotla et al., 2020). However, this study is one of the first to observe the effect of SKY intervention on HCPs during COVID-19.

This study observed the impact of SKY on HCPs working in COVID-19 care units, where the stress was at its peak. Overall, the results of this study for different assessed parameters suggest that SKY has a significant and positive impact on HCPs’ mental health and work engagement.

### Professional Fulfillment

Healthcare professionals’ Professional Fulfillment was adversely affected during COVID-19 (O’Brien et al., 2021). As perceived by Sarma et al. (2020), even HCPs working in non-COVID areas faced challenges. For instance, the risk of infection to themselves and their families, the lack of any concrete protocol for patient management, reduced staff availability, delay in discharging duties toward their patients, and increased workload. The pandemic affected their physical and mental well-being and work-life balance. A study conducted during COVID-19 found significantly lower Professional Fulfillment in HCPs, irrespective of their exposure to COVID-19 (Kannampallil et al., 2020). Another study conducted between July 2019 and 1 March 2020 found that burnout and Professional Fulfillment were present in 35 and 58% of medical critical care physician responses, respectively. In comparison, during the COVID-19 pandemic, burnout and Professional Fulfillment were present in 57 and 38%, respectively (Gomez et al., 2020). In the current study, a significant improvement was seen in the Professional Fulfillment experienced by the HCPs who practiced SKY compared to those in the Control Group who did not practice any mind-body intervention. The study results indicate that Professional Fulfillment continued to increase among the HCPs practicing SKY for 30 days, despite experiencing greater work exhaustion during the same period (as shown by scores on day 30 assessment). Recently, one study found that yoga helps in improving Professional Fulfillment among HCPs. In the study, HCPs’ Professional Fulfillment increased from 8.3% at baseline to 27.3% post-yoga program (Scheid et al., 2020). Interoceptive awareness reduces the risk of burnout, and for higher interoceptive awareness, yoga-based meditation is feasible and acceptable for HCPs, according to Lehto et al. (2020). A study on SKY revealed that it improved the quality of sleep, enhanced life satisfaction, and increased resilience during the COVID-19 pandemic among HCPs (Divya et al., 2021). Here, the study results indicate that SKY helped HCPs increase Professional Fulfillment through self-worth, satisfaction, and a feeling of contribution to SKY, which is also evident from the study conducted, even while dealing with the increasing COVID-19 cases at work and experiencing greater work exhaustion.

This study is the first to examine the impact of SKY on Professional Fulfillment. It provides elemental evidence for the significant improvement in Professional Fulfillment among HCPs with SKY practice despite greater work exhaustion.

### Work Exhaustion

Work exhaustion refers to work-related stress or burnout and includes both physical and emotional exhaustion. Stress is experienced when we have too much to do and too little energy or time. For HCPs, the burden of patient care, long and tiring work hours, and inadequate availability of effective medications or treatments are significant factors contributing to stress.

Bridgeman et al. (2018) found that burnout syndrome was progressively recognized among HCPs even before the pandemic. During the COVID-19 pandemic, it increased significantly due to heightened workload as well as the time spent with patients for ongoing explanations and comfort, adding more responsibility and pressure to HCPs’ work (Barello et al., 2020; Khasne et al., 2020; Morgantini et al., 2020; Conti et al., 2021; Jalili et al., 2021; Martínez-López et al., 2021; O’Brien et al., 2021).

Research studies on SKY have found it useful in relieving stress, anxiety, major depression, and mood disorders (Kjellgren et al., 2007; Doria et al., 2015; Goldstein et al., 2020). SKY has also shown a reduction in cardiovascular risks in patients with anxiety and depression disorders (Brown et al., 2005; Viveka et al., 2013; Toschi-Dias et al., 2017).

Both brain and heart functions affect mental stress. SKY plays a significant role in effectively reducing mental stress, improving human stress tolerance and enhancing cognitive performance (Chandra et al., 2017). It was noted from the current study results that work exhaustion was significantly reduced in the Experimental Group compared to the Control Group after the SKY session and practice. Work exhaustion was decreased immediately after SKY among HCPs during COVID-19. It is possible that the effects were reduced after 30 days due to the escalating COVID-19 cases in India. Even though work exhaustion increased, the positive attitude and interpersonal disengagement continued to improve after SKY practice.

### Interpersonal Disengagement

Interpersonal disengagement is the withdrawal from social interaction. According to Thanacoody et al. (2013), emotional exhaustion leads to interpersonal disengagement and, in turn, negatively impacts affective commitment and the turnover intentions of HCPs toward their work. The greater the exhaustion, the greater the risk of disengagement. During the Pandemic, a study found that 53.8% of the HCPs were emotionally exhausted, and 35.1% were found to be suffering from depersonalization (Martínez-López et al., 2021). Similar results were obtained in another study on the impact of SKY on social work, which revealed that SKY brings about feelings of responsibility toward society (Pandya, 2015). These study results indicate that SKY practice reduced interpersonal disengagement among HCPs during COVID-19, even though the differences were not statistically significant. A study on yoga as an intervention indicated that yoga significantly improves interpersonal disengagement among HCPs (Scheid et al., 2020). The reduction was particularly notable on Day 30 of the SKY practice. Contradicting Thanacoody et al. (2013), who say that the greater the exhaustion, the greater the risk of disengagement, here, it was found that even though work exhaustion was noticeable on Day 30 compared to the immediate effect of SKY, interpersonal disengagement reduced significantly on Day 30 in the SKY group (Table 6). This is in accordance with the findings of a study by Chandra et al. (2017), which found that SKY helps with stress endurance.

### Positive and Negative Affect

After practicing SKY, HCPs scored higher for PA, suggesting that they were full of energy and enthusiasm. The NA also reduced, showing a reduction in sadness and lethargy and increased mental peace. Study results indicate that SKY helped HCPs improve their mood and have a positive outlook. It also helped alleviate the response to negative experiences. Previous research has demonstrated that SKY helps manage emotions and feelings (Kjellgren et al., 2007; Goldstein and Uchida, 2016), which is also evident here. PA and NA scores improved immediately after the SKY intervention and during the 30-day assessment. The comparison between the Experimental and Control Groups showed the effectiveness of SKY. Using the PANAS scale, La Torre et al. (2020) studied the effect yoga and mindfulness had; they found a statistically significant improvement in the NA domain but not in the PA domain after yoga. However, SKY was found to improve both.

There have been an inadequate number of studies assessing the impact of yoga and meditation practices among HCPs during COVID-19. The study done by Klatt et al. (2020) suggested embracing mindfulness to reduce stress and burnout and increase resilience and work engagement for HCP faculty and staff during the COVID-19 pandemic. Dzau et al. (2020) and Olson et al. (2019) suggested the integration of wellness programs and initiatives to reduce burnout and improve Professional Fulfillment among the healthcare workforce during the COVID-19 pandemic. SKY can be explored as an addition to such wellness programs. This study was attempted in a pandemic situation. Further studies on the impact of SKY among HCPs should be conducted with a bigger sample size and better study techniques to establish the study results. The long-term effects of SKY on participants could also be studied on Day 180 post-intervention. Despite the small sample size, the results of this study provide strong evidence to bolster the use of yoga as a way for HCPs to boost their well-being.

### Limitations

The findings of this study have certain limitations. The number of doctors practicing in the study field during the specified period from July 2020 to October 2020 was the study sample that restricted the study to a small sample size. Given their busy schedules, time constraints were the greatest challenge to engaging HCPs in a study during a crisis such as the COVID-19 pandemic. This hampered the ability to conduct detailed demographic assessments, resulting in four dropouts in the Experimental Group on Day 30. Social distancing and lockdown further restricted this study and required the study intervention to be delivered online. However, this was an opportunity for exploratory rather than an explanatory research design, which had to overlook randomization, active control, recall bias, non-respondent biases, and explanations to rule out alternatives in methodologies. There is scope for further research into multicentric research.

## Conclusion

Although many research studies have been conducted globally to observe the impact of the COVID-19 pandemic on mental health, stress, and work exhaustion among HCPs, very few have focused on investigating the efficacy of yogic interventions as an option to reduce stress and work exhaustion while also improving mental health in HCPs during the pandemic.

In this unique pilot study, it was found that SKY can aid HCPs in maintaining their well-being when confronted with unprecedented challenges. SKY elevated Professional Fulfillment (*p* = 0.04) immediately after the intervention among HCPs compared to their colleagues in the Control Group. The SKY effect was maintained on Professional Fulfillment, even after 30 days of continued SKY practice (*p* = 0.02). Findings also indicate that, as compared to the Control Group, SKY helped reduce work exhaustion (*p* = 0.01) and the negative impact (*p* < 0.001) of the pandemic immediately after the intervention. However, within the Experimental Group, the levels of work exhaustion were found to have increased on Day 30 of SKY practice. Intriguingly, their 30. SKY helped HCPs increase Professional Fulfillment, reducing the negative interpersonal disengagement that was not affected by increased work exhaustion. In fact, the levels of interpersonal disengagement significantly reduced (*p* = 0.05) on Day 30 impact and interpersonal disengagement and handled work exhaustion during the peak of the pandemic.

## Data Availability Statement

The raw data supporting the conclusions of this article will be made available by the authors, without undue reservation.

## Ethics Statement

The studies involving human participants were reviewed and approved by the Medanta Institutional Ethics Committee (reference number MICR-1106/2020). The patients/participants provided their written informed consent to participate in this study.

## Author Contributions

All authors made significant contributions, reviewed and approved the final version of the manuscript.

## Conflict of Interest

The authors declare that the research was conducted in the absence of any commercial or financial relationships that could be construed as a potential conflict of interest.

## Publisher’s Note

All claims expressed in this article are solely those of the authors and do not necessarily represent those of their affiliated organizations, or those of the publisher, the editors and the reviewers. Any product that may be evaluated in this article, or claim that may be made by its manufacturer, is not guaranteed or endorsed by the publisher.
