# Supplementary material for: Alleviating Work Exhaustion, Improving Professional Fulfillment, and Influencing Positivity Among Healthcare Professionals During COVID-19: A Study on Sudarshan Kriya Yoga
Source: Front Psychol. 2022 Jul 13;13:670227. doi: 10.3389/fpsyg.2022.670227 (PMC9326464; doi:10.3389/fpsyg.2022.670227)
Supplement: Supplementary file 1 [file Table_1.docx]

| **Table 1: Participant's Characteristics at baseline for Experimental Group (N=29) and Control Group (N=27)** | | | |
| --- | --- | --- | --- |
| Characteristics | Type | All Participants Experimental Group | All Participants Control Group |
| Gender n(%) | Male | 10(34.50) | 10(37.00) |
|  | Female | 19(65.50) | 17(63.00) |
| Age | Mean(SD) | 37.1 (9.90) | 28.5(7.30) |
|  | Min | 25 | 21 |
|  | Max | 66 | 47 |
