# Supplementary material for: Alleviating Work Exhaustion, Improving Professional Fulfillment, and Influencing Positivity Among Healthcare Professionals During COVID-19: A Study on Sudarshan Kriya Yoga
Source: Front Psychol. 2022 Jul 13;13:670227. doi: 10.3389/fpsyg.2022.670227 (PMC9326464; doi:10.3389/fpsyg.2022.670227)
Supplement: Supplementary file 2 [file Table_2.docx]

| **Table 2(a): Average Values (Standard Deviation) comparison between Experimental and Control group before the intervention**  *p values <0.05 & ** p value <0.01 | | | |
| --- | --- | --- | --- |
|  | **PRE Experimental** | **PRE Control** | **Pre Experimental_Pre Control** |
|  | Mean (SD) | Mean (SD) | p Value |
| **Professional Fulfillment** | 17.48 (5.24) | 14.96(6.10) | 0.28 |
| **Work Exhaustion** | 5.16 (3.70) | 5.81 (3.64) | 0.44 |
| **Interpersonal Disengagement** | 5.00 (5.67) | 4.40 (5.38) | 0.94 |
| **PANAS Positive** | 36.92 (9.08) | 36.33 (9.22) | 0.84 |
| **PANAS Negative** | 19.80 (7.12) | 24.22 (7.88) | 0.06 |

p values are based on two-way MANOVA at the significance level of 0.05

| **Table 2(b): Average Values (Standard Deviation) comparison between Experimental and Control group after the intervention**  *p values <0.05 & ** p value <0.01 | | | |
| --- | --- | --- | --- |
|  | **POST Experimental** | **POST Control** | **Post Experimental_Post Control** |
|  | Mean (SD) | Mean (SD) | p Value |
| **Professional Fulfillment** | 19.12 (4.87) | 14.96 (6.10) | 0.04* |
| **Work Exhaustion** | 3.28 (2.91) | 5.81 (3.64) | 0.01* |
| **Interpersonal Disengagement** | 3.96 (6.40) | 4.4 (5.38) | 0.62 |
| **PANAS Positive** | 41.88 (7.20) | 36.33 (9.22) | 0.05* |
| **PANAS Negative** | 15.52 (7.89) | 24.22 (7.88) | 0.00** |

p values are based on two-way MANOVA at the significance level of 0.05, * p values <0.005 & ** p value <0.001

| **Table 2(c): Average Values (Standard Deviation) comparison between Experimental and Control group 30 days after the intervention**  *p values <0.05 & ** p value <0.01 | | | |
| --- | --- | --- | --- |
|  | **Day 30 Experimental** | **Day30 Control** | **Day 30 Experimental_Day 30 Control** |
|  | Mean (SD) | Mean (SD) | p values |
| **Professional Fulfillment** | 19.00 (5.12) | 14.96 (6.10) | 0.02* |
| **Work Exhaustion** | 4.52 (3.86) | 5.81 (3.64) | 0.20 |
| **Interpersonal Disengagement** | 2.36 (3.35) | 4.40 (5.38) | 0.05* |
| **PANAS Positive** | 41.60 (7.14) | 36.33 (9.22) | 0.05* |
| **PANAS Negative** | 14.96 (6.06) | 24.22 (7.88) | 0.00** |

p values are based on two-way MANOVA at the significance level of 0.05, * p values <0.005 & ** p value <0.001
