# Supplementary material for: Alleviating Work Exhaustion, Improving Professional Fulfillment, and Influencing Positivity Among Healthcare Professionals During COVID-19: A Study on Sudarshan Kriya Yoga
Source: Front Psychol. 2022 Jul 13;13:670227. doi: 10.3389/fpsyg.2022.670227 (PMC9326464; doi:10.3389/fpsyg.2022.670227)
Supplement: Supplementary file 3 [file Table_3.docx]

| **Table 3: Effect size in Experimental Group** | | |
| --- | --- | --- |
|  | **Cohen’s d value** | |
|  | **Pre_Post** | **Pre_Day 30** |
| **Professional Fulfillment** | -0.36 | -0.32 |
| **Work Exhaustion** | 0.51 | 0.14 |
| **Interpersonal Disengagement** | 0.15 | 0.53 |
| **PANAS Positive** | -0.62 | -0.50 |
| **PANAS Negative** | 0.60 | 0.70 |
