# Supplementary material for: Alleviating Work Exhaustion, Improving Professional Fulfillment, and Influencing Positivity Among Healthcare Professionals During COVID-19: A Study on Sudarshan Kriya Yoga
Source: Front Psychol. 2022 Jul 13;13:670227. doi: 10.3389/fpsyg.2022.670227 (PMC9326464; doi:10.3389/fpsyg.2022.670227)
Supplement: Supplementary file 4 [file Table_4.docx]

| **Table 4: Average Values (Standard Deviation) in the Experimental Group**  *p values <0.05 & ** p value <0.01 | | | | | |
| --- | --- | --- | --- | --- | --- |
|  | **PRE Experimental** | **POST Experimental** | **Day 30 Experimental** | **p Values Experimental** | |
|  | Mean(SD) | Mean(SD) | Mean | Pre_Post | Pre_Day 30 |
| **Professional Fulfillment** | 17.50 (5.1) | 19.20 (4.7) | 19.00 (5.0) | 0.06 | 0.10 |
| **Work Exhaustion** | 5.00 (3.6) | 3.30 (3.0) | 4.60 (3.8) | 0.01** | 0.20 |
| **Interpersonal Disengagement** | 4.60 (5.4) | 3.72 (6.1) | 2.60 (3.5) | 0.51 | 0.02* |
| **PANAS Positive** | 37.70 (8.8) | 42.60 (7.0) | 41.10 (7.3) | 0.01* | 0.07 |
| **PANAS Negative** | 22.3 (8.2) | 17.20 (8.8) | 17.0 (6.7) | 0.00** | 0.00** |

p values are based on paired sample t test at the significance level of 0.05, * p values <0.005 & ** p value <0.001
