# Supplementary material for: Alleviating Work Exhaustion, Improving Professional Fulfillment, and Influencing Positivity Among Healthcare Professionals During COVID-19: A Study on Sudarshan Kriya Yoga
Source: Front Psychol. 2022 Jul 13;13:670227. doi: 10.3389/fpsyg.2022.670227 (PMC9326464; doi:10.3389/fpsyg.2022.670227)
Supplement: Supplementary file 5 [file Table_5.docx]

| **Table 5: Correlation Coefficient and Probability Value among Experimental Group** | | | | | | |
| --- | --- | --- | --- | --- | --- | --- |
|  | **PRE** | | **POST** | | **Day 30** | |
| **Correlation** | **Correlation coefficient** | **p value** | **Correlation coefficient** | **p value** | **Correlation coefficient** | **p value** |
| Professional Fulfillment & Work Exhaustion | -0.4 | 0.05* | -0.5 | 0.01** | -0.3 | 0.17 |
| Professional Fulfillment & Interpersonal Disengagement | -0.2 | 0.39 | -0.3 | 0.07 | -0.4 | 0.03* |
| Professional Fulfillment & PANAS Positive | 0.5 | 0.01** | 0.7 | 0.00** | 0.3 | 0.18 |
| Professional Fulfillment & PANAS Negative | -0.1 | 0.53 | -0.4 | 0.03* | -0.0 | 0.84 |
| Work Exhaustion & Interpersonal Disengagement | 0.8 | 0.00** | 0.5 | 0.01* | 0.4 | 0.03* |
| Work Exhaustion & PANAS Positive | -0.3 | 0.14 | -0.2 | 0.41 | -0.1 | 0.64 |
| Work Exhaustion & PANAS Negative | 0.3 | 0.10 | 0.8 | 0.00** | 0.4 | 0.05* |
| Interpersonal Disengagement & PANAS Positive | -0.3 | 0.11 | -0.1 | 0.79 | -0.4 | 0.07 |
| Interpersonal Disengagement & PANAS Negative | 0.4 | 0.03* | 0.6 | 0.00** | 0.3 | 0.12 |
| PANAS Positive & PANAS Negative | -0.1 | 0.60 | -0.0 | 0.85 | -0.0 | 0.86 |

*. Correlation is significant at the 0.05 level (2-tailed).

**. Correlation is significant at the 0.01 level (2-tailed).
