# Supplementary material for: Alleviating Work Exhaustion, Improving Professional Fulfillment, and Influencing Positivity Among Healthcare Professionals During COVID-19: A Study on Sudarshan Kriya Yoga
Source: Front Psychol. 2022 Jul 13;13:670227. doi: 10.3389/fpsyg.2022.670227 (PMC9326464; doi:10.3389/fpsyg.2022.670227)
Supplement: Supplementary file 6 [file Table_6.docx]

| **Table 6: Cronbach’s Alpha** | | | |
| --- | --- | --- | --- |
| **Scale** | **Pre** | **Post** | **Day 30** |
| Professional Fulfillment | 0.89 | 0.91 | 0.94 |
| Work Exhaustion | 0.76 | 0.74 | 0.84 |
| Interpersonal Disengagement | 0.91 | 0.97 | 0.87 |
| PANAS Positive | 0.93 | 0.93 | 0.91 |
| PANAS Negative | 0.91 | 0.92 | 0.87 |
